# Supplementary material for: Meta-Reinforcement Learning reconciles surprise, value, and control in the anterior cingulate cortex
Source: PLoS Comput Biol. 2025 Apr 22;21(4):e1013025. doi: 10.1371/journal.pcbi.1013025 (PMC12047761; doi:10.1371/journal.pcbi.1013025)
Supplement: S1 Text — (DOCX) [file pcbi.1013025.s001.docx]

**Supplementary material**

**Meta-Reinforcement Learning reconciles surprise, value, and control in the anterior cingulate cortex.**

Tim Vriens, Eliana Vassena, Giovanni Pezzulo, Gianluca Baldassarre & Massimo Silvetti

**1. Supplementary Methods**

- 1. **The RML**

In this study, we model decision-making using a computational model, the reinforcement meta-learner (RML(1)). While already summarized in the main text, this section aims to expand on the description of the RML, and use formal descriptions of the processes described in the text. As the aim is to provide an explanation that can be read without prior knowledge on this model, several of the formulas provided in this document come from an earlier study describing the RML(2). For any further clarifications, we will refer to this paper.

As a reinforcement learning algorithm, the RML models decision-making as a Markov decision process in discrete time. It assumes that the environment over time can be split in a sequence of states, denoted by *s*. The state at a certain timepoint *t* is denoted by *s_t_*. In each of these states, the agent is prompted to select one of several different actions. Each of these actions, denoted by *a*, all have action-outcome contingencies. Each action will result in both a state transition to state *s’*, and a direct reward, denoted by *r*. We note that this direct reward can be zero, meaning that an action only changes the current state of the environment, without yielding a reward. Importantly, the model does not know these contingencies at the start of the task: they have to be learned.

The RML models the action selection and learning as two distinct parts of an action. Once a state is observed, an action has to be taken, and only after this action is taken, and the outcome is received, can the value of this action be updated.

The RML has several features distinguishing it from other reinforcement learning paradigms. First, it takes two dimensions of actions into account. Their expected value, and the amount of effort – be it physical or mental – required to execute this action. In order to select the most optimal value, the reward received from the action is discounted by the effort required to obtain it. The optimal action is then selected using a softmax function.

A second feature is its ability to regulate several of its own internal parameters. For one, the RML regulates the extent to which the reward is discounted by the effort. Additionally, the reward sizes are regulated.

Thirdly, the RML incorporates meta-learning; it is able to learn about the learning itself. The RML is able to optimize its own learning rate based on the volatility of the environment, enabling the agent to optimize the speed at which it learns.

The RML performs decision-making using four different modules. It uses a **boost module**, an **action selection module**, a **feedback module**, and an **control module**(1). Both the **boost module** and **action selection module** perform action selection in a way similar to an actor module in traditional Q-learning algorithms, with the difference that they integrate an adaptive learning rate. The **boost module** regulates the optimization of the effort discounting and reward size parameter. It does so by selecting a boost intensity, indicating to what extend the model should upregulate its internal parameters. Critically, this upregulation of parameters is costly. The higher the effort discounting and reward size parameter, the higher the cost of the boosting action. The **action selection module** selects the most optimal action to be executed by the agent. The **feedback module** processes primary and non-primary reward from the environment, and uses the signal from the boost module to modulate the total reward experienced during the trial. This reward is subsequently provided to the boost module and **action selection module** to learn about the outcome of their selected actions. The **control module** integrates the signal from the **boost module,** discounting the expected effort required to perform each action, thereby acting as an interface between the **boost module** and **action selection module.**

In the next part, we will first explain the equations used by the RML to optimize decisions. Afterwards, we give a detailed description on the neural structure underlying the decision-making process by the RML.

**1.2 Action selection**

The RML models action selection in two steps. First, the optimal intensity of boosting is selected. This boosting has two functions: it regulates the effort discounting and reward size parameter, as detailed below. This is performed by the **boost module**. The **boost module** selects the optimal boosting using the perceived environmental state. In the RML, this intensity of boosting is discrete: several predetermined intensities are available to be selected. This allows for the boost module to work in a way similar to a Q-learning actor, where the possible actions are the intensities of boosting available (the intensity of boosting at timepoint *t* is denoted by *b_t_*). When faced with an environmental state, the boost module selects a value for *b_t_* by evaluating the expected reward for each option from a stored table denoted by *V_boost_*(*b_t_*). Then, the realized boost intensity is selected using a softmax function (see Equation S1).

This intensity of boosting does not directly influence the **action selection module**. First, the **boost module** sends the signal for the selected intensity of boosting to the **control module**. The **control module** then transforms this *boost* signal (see Equation S2). This transformed value is sent to the **action selection module**, where it is used to make a decision about which action to select. To this end, the **action selection module** considers both the current state *s_t_*, the possible actions (*a_1_, …, a_n_*), and the learned action values (denoted by *V_act_*(*a_t_*)). It selects an action using a softmax, as described in Equation S3. The RML can be connected to external, task-specific modules, here referred as **external module**. This external module can be influenced by the activity in the **control module** and the expected value of the different actions, and in turn influence the decisions made by the RML by affecting the **action module**.

**1.3 Learning**

After this action is selected and executed, the agent receives feedback from the environment. This feedback can come in two different forms: a reward (denoted by *R*), and a transition to a different state (denoted by *s’*). When this feedback is received, the **feedback module** processes this feedback into an experienced value linked to both primary and non-primary reward. This value signal is then directed to both the **action selection module** (denoted by *DA_act_*, see Equation S4a) and to the **boost module** (denoted by *DA_boost_*, see Equation S4b). The output of the **feedback module** does not depend only on the environment, but it is modulated by the *boost* signal in the following ways.

For the **action selection module**, the experienced value is upregulated by the boost signal selected during the action selection. In the case of the **boost module**, the experienced reward is discounted by the cost of boosting, which is linearly dependent on the boost intensity *b*.

The model uses these experienced values to calculate the prediction error (PE), the difference between the expected value and the experienced value. This PE is then multiplied by an adaptive learning rate and then used to update the expected values for the selected action *a* (see Equation S5a) and boost intensity *b* (see Equation S5b).

**1.4 Dynamic learning rate**

Differently from classical Q-learning, the learning rate for the **boost module** and **action selection module** is adaptive. The learning rate is a function of the ratio of the variance due to environmental changes (volatility) to the variance due to random fluctuations (noise). This means that environmental changes increase plasticity while random fluctuations decrease it. This calculation can be modeled using an approximation of a **Kalman filter**(3) (equations S5-6). For this reason, the product of the PE by the adaptive learning rate (which determines the Q value update) is equivalent to an approximated Bayesian surprise.

**1.5 RML-to-brain mapping**

The RML is a bio-inspired system based on the meta-reinforcement learning framework proposed by Doya et al.(4) It is inspired by previously identified bidirectional connections between different neural structures, specifically the medial prefrontal cortex (MPFC), locus coeruleus (LC), and ventral tegmental area (VTA) (e.g. 4-6). The combination of the **action selection module** and **boost module** are hypothesized to simulate MPFC, including the dorsal anterior cingulate cortex (dACC)(1,2). These modules are bidirectionally connected with the **control module** and the **feedback module**, which are represented by the catecholamine brainstem nuclei. Specifically, the **control module** simulates the LC, while the **feedback module** simulates the VTA. The latter generates a dopamine (DA) signal, which provides an experienced reward signal to the **action selection module** and the **boost module**. The effort discounting parameter is signaled by the LC, by a generated noradrenaline (NE) signal. Additionally, we hypothesize that also the adaptive learning rate is computed in the LC(8). See Fig 1A and 1B in the main text for a graphical representation of the anatomical representation and connections of the RML.

**1.6 RML Equations**

| $p\left( selected boost= b \vert s \right)=\sigma(v_{\mathrm{boost}}(s,b),\tau_{boost})$ | (S1) |
| --- | --- |

This equation defines the probability *p* of selecting boost level *b* while in state *s*. $v_{boost}\left( s,b \right)$ is the expected value of boost level *b* given the current state *s*. $\tau_{boost}$ indicates the temperature of the softmax function *σ*. Here, *b* is a natural number, such that $1\leq b\leq10$.

|  | $NE_{LC}(b)=f(b)$ | (S2) |
| --- | --- | --- |

In this equation, $NE_{LC}(b)$ is the LC signal (i.e. the LC-generated *NE* signal), which is set as a function dependent on the boost level *b*. In the paper by Silvetti et al.(1) describing the RML, this formula is set to be the identity, for the sake of simplicity. Therefore, the value of $NE_{LC}(b)$ is set to be equal to the boost value.

|  | $p\left( chosen action=a \vert s \right)=\sigma(v_{act,t}\left( s,a \right)-\frac{C\left( s,a \right)}{NE_{LC}\left( b \right)},\tau_{act})$ | (S3) |
| --- | --- | --- |

This equation determines the probability *p* of selecting a certain action *a,* directed toward the environment, given state *s*. $v_{act,t}\left( s,a \right)$ is the learned value of action *a* given state *s*. $\tau_{act}$ indicates the temperature of the softmax function *σ*. $C\left( s,a \right)$ is the cost value of action *a* given state *s*.

|  | $DA_{act,t}=(r_{t}\left( R_{t}+\mu b \right)+b\left( 1-\mu\right)\rho\max_{a\epsilon A_{s'}} (v_{act,t}(s',a)))$ | (S4a) |
| --- | --- | --- |
|  |  |  |
|  | $DA_{boost,t}=r_{t}R_{t}-\omega b+\max_{b\epsilon B_{s'}} (v_{boost,t}(s',b)))$ | (S4b) |

Equations S4a and S4b show the calculation of the VTA signals (i.e. the VTA-generated DA signals) for both the action (S4a) and boost (S4b) module. For the action module, this signal is a combination of the presence (*r_t_*) and size (*R_t_*) of the reward at time *t*, with an added value depending on the amount of cognitive control, in case a reward was present. This cognitive control is multiplied by a meta-parameter of the system governing the DA dynamics, 𝜇. Furthermore, the most optimal future reward (i.e. the maximum reward of any action in the next state) is taken into account, multiplied with a temporal discounting parameter 𝜌. In the equation, *A_s’_* is the collection of all possible actions given state *s’*.

For the boost module, the VTA signal is defined as the actual reward received in the trial, where the cost of the boosting is subtracted. The cost of the boosting is equal to the level of boosting, multiplied with $\omega$, a parameter describing the cost of boosting. The higher this parameter, the more costly it is to perform boosting. Additionally, the boost module also looks at the most optimal future reward by estimating the optimal reward in the next state.

|  | $\Delta v_{act,t}\left( s,a \right)=\lambda_{act,t}\cdot{(DA_{act,t}-v}_{act,t-1}\left( s,a \right))$ | (S5a) |
| --- | --- | --- |
|  |  |  |
|  | ${\Delta v}_{boost,t}\left( s,b \right)=\lambda_{boost,t}\cdot{(DA_{boost,t}-v}_{boost,t-1}\left( s,b \right))$ | (S5b) |

Equations S5a and S5b show the update of the learned expected state-action value after a trial. The learning system uses the dynamically changing learning rate ($\lambda_{act,t}$ and $\lambda_{boost,t}$ respectively), as calculated in Equation S6, as well as the experienced value of the trial at time *t*, conveyed by the VTA-generated DA signal (${DA}_{act,t}$ and ${DA}_{boost,t}$ respectively) to update its internal value. After calculating the update value ($\Delta v_{act,t}\left( s,a \right)$ or $\Delta v_{boost,t}\left( s,b \right)$), the expected value is updated by adding the update value.

|  | $\lambda_{act,t}=max(\frac{\hat{Var_{t}}\left( v \right)}{{\hat{\delta_{t}}}^{2}},\beta)$ | (S6) |
| --- | --- | --- |

This equation calculates the learning rate for the action module as the quotient between the expected variance of *v* at timestep *t* ($\hat{Var_{t}}\left( v \right),$calculated in Equation S7), and the squared prediction error at timestep *t* (${\hat{\delta}_{t}}^{2}$, calculated in Equation S9). If this value is smaller than $\beta$, the learning rate is set to $\beta$ to avoid computational instability. As described in previous papers(1,2), the equations S6-S9 approximate a Kalman gain(3).

|  | $\hat{Var_{t}}\left( v \right)=\left( v_{t}-\hat{v}_{t-1} \right)^{2}$ | (S7) |
| --- | --- | --- |

Equation S7 describes the generation of the expected variance of *v* at time *t* ($\hat{Var_{t}}\left( v \right)$), as a function of the timecourse of the value $v_{t}$and the time-smoothed value at the previous timestep ($\hat{v}_{t-1}$), calculated in Equation S8.

|  | $\hat{v}_{t}=\hat{v}_{t-1}+\alpha\cdot(v_{t}-\hat{v}_{t-1})$ | (S8) |
| --- | --- | --- |

Equation S8 describes the timecourse of the time-smoothed value $\hat{v}_{t}$: each timestep, it updates as a combination of the old value of the time-smoothed value, and the difference between the current value of the trial and the old time-smoothed value, with a weight of $\alpha$. This $\alpha$ is a parameter determining the amount of smoothing in the time-smoothed value, as well as the amount of smoothing in the time-smoothed prediction error (as shown in Equation S9).

|  | $\hat{\delta}_{t}=\hat{\delta}_{t-1}+\alpha\cdot({\vert\delta}_{t}\vert-\hat{\delta}_{t-1})$ | (S9) |
| --- | --- | --- |

Equation S9 shows the calculation of the time-smoothed prediction error ($\hat{\delta}_{t}$), which is defined similarly to the time-smoothed value in Equation S8. It is a combination of the old value of the time-smoothed prediction error, combined with the difference between the absolute value of the current prediction error and the old value of the time-smoothed prediction error, with a weight $\alpha$.

| Free parameters | | | |
| --- | --- | --- | --- |
| Variable | Description | Equation | Value |
| $\tau_{boost}$ | Temperature of the softmax for the boost module | S1 | 0.3 |
| $\tau_{act}$* | Temperature of the softmax for the action selection module | S3 | N.A. |
| 𝜇 | Parameter indicating DA dynamics | S4a | 0.1 |
| $\rho$ | Temporal discounting parameter | S4a | 0.1 |
| $\alpha$ | Temporal smoothing parameter in determining learning rates | S8, S9 | 0.3 |
| $\beta$ | Minimum learning rate | S6 | 0.15 |

***Table A****: Free parameters used in the RML, equations where each parameter is used. These parameters are fixed in the current study and are inherited from*(1)*.*

- 1. **Speeded decision-making task modeling**

In order to model the task by Vassena et al.(9) using the RML, we used the drift diffusion model (DDM) as task-specific module, and replicated the results by using a dual attractor model (DAN). The task consisted of 36 possible states. Each of these states consisted of a two-option decision-making task. The left and right options were independently set to yield a reward equal to an integer between 2 and 7. All possible combinations of the left and right reward were considered. Each state had an intrinsic difficulty, defined by the difference between the reward received from the left and right reward. We modeled the task difficulty inversely linear to the difference between the expected value of the left and right option; the more similar both options are, the more difficult it is to make a decision between them. Each trial had a response deadline (14000 DDM cycles), that, if exceeded, resulted in the trial termination with no reward delivered.

Before modeling the task, the RML is given a prior value for each set of fractals, equal to the actual value of the fractal. Then, the RML is given an explorative training, where it explores all options and decisions, in order to optimize the prior value for each set of fractals. Afterwards, the RML performs 1944 trials (54 trials per state). During the analysis, these states are sorted by value difference (the difference between the left and right option), and the mean boost (Eq. S1) and sum of the mean expected values of the boost and actions (Eq. S5a,b) are plotted. The total dACC activity was set to the sum of the normalized (divisive normalization for the DDM and z-scoring for the DAN) mean boost and mean value. In total, 200 simulations were performed, and the average results were reported.

**1.8 The drift diffusion model**

The drift diffusion model (DDM) was first introduced by Ratcliff(10). It models a decision between two options by keeping track of a confidence variable, indicating both which option the agent is currently favoring, and to what extend the agent is confident about its choice. The model accumulates its confidence over time, until a preset decision boundary is reached. At that point, it selects the option corresponding to the decision boundary reached.

Mathematically, the confidence generated during each timestep is a combination of the drift rate (indicating the average confidence gain due to the environment, denoted by *DR* in this paper to avoid confusion with the variable indicating value in the RML) and random Gaussian noise (with a mean of 0, and standard deviation of *σ*). Normally, the DDM uses two decision boundaries. One occurs when the accumulated confidence value reaches a threshold level (denoted by $\theta$’), and the other when it reaches 0. In an unbiased version of the DDM, the accumulated confidence starts at a value of $0.5\cdot\theta$’ at the beginning of each trial. For simplicity, we make a small notational change to this model. Instead of starting at $0.5\cdot\theta$’, and performing a decision at either 0 or $\theta$’, we let the model start at 0, and make a decision when the confidence reaches either - $\theta$ or $\theta$, where $\theta$ is equivalent to $0.5\cdot\theta$’.

In our combination, we combine the RML with the DDM to gain two different outputs. First, we are interested in which decision the DDM makes, indicated by the decision boundary reached by the confidence variable. Additionally, we are interested in the duration it took to reach a decision, which can be used to determine the reaction time of the trial.

**1.9 RML-DDM interface**

In order to combine the DDM with the RML, several RML values are used as input in the DDM (see Fig 2A from the main text for a visual overview of the connection between the DDM and the RML). Specifically, the value of the drift rate and threshold level both depend on RML parameters. The drift rate is directly proportional to the expected difficulty of the trial, the difference in the expected value of one option compared to the other. It is set according to Equation S10:

| $DR=v_{DDM}\cdot(v_{act}\left( a_{1},s \right)-v_{act}(a_{2},s))$ | (S10) |
| --- | --- |

Here, *DR* is the drift rate, $v_{DDM}$ is a model parameter that determines the influence of the difficulty on the drift rate. $v_{act}\left( a_{1},s \right)$ and $v_{act}\left( a_{2},s \right)$ denote the estimated value selecting action 1 or action 2 in the current state *s* as computed by the RML respectively.

Additionally, the initial threshold is set to the quotient of the NE level and a model parameter, scaling this boost level for use in the DDM, as shown in Equation S11:

| $\theta=\theta_{DDM}/{NE}_{LC}$ | (S11) |
| --- | --- |

Here, $\theta_{DDM}$ is a model parameter, while *NE_LC_*  is from Equation S2.

The DDM starts by setting the confidence value (denoted by *c*(*t_TR_*), a function of *t_TR_*, the time passed within the trial) to 0, at time *t_TR_*=0 and evolves over time following Equation S12. A decision is made, (at time *t_TR_*>0), when *c*(*t_TR_*) is either equal to θ, or – θ. Additionally, in case the model has not reached either decision threshold when the time limit (14000 DDM cycles) is reached, the model is set to be too late in making a decision. In this case, no reward was given in that trial.

| $c\left( t_{TR}+dt_{TR} \right)=c\left( t_{TR} \right)+\left( DR+N\left( 0,\sigma_{DDM} \right) \right)\cdot dt_{TR}$ | (S12) |
| --- | --- |

This equation describes the evolution of *c* over time. In this equation, *DR* denotes the drift rate for the current trial (calculated according to Equation S10), and $N\left( 0,\sigma_{DDM} \right)$ denotes random noise drawn from a normal distribution with mean 0 and standard deviation $\sigma_{DDM}$, a model parameter of the DDM. In the simulations, $dt_{TR}$ is set to 0.01.

The DDM then provides the RML with both the decision made and the value of $t_{TR}$ at which this decision is made. This value of $t_{TR}$ is used to get the reaction time. In order to ensure the RML optimizes its decisions to optimize both accuracy and reaction time, the model receives a reward penalty depending on its reaction time (see Equation S13). The slower the agent responds, the larger this penalty is.

| $R=RW_{act}-\frac{RT_{DDM}}{v_{RT}}$ | (S13) |
| --- | --- |

In this equation, the trial reward (*R*) is a function of the received reward due to the chosen action ($RW_{act}$), and the penalty based on the reaction time. This penalty is given as the quotient between the found reaction time of this trial due to making a decision according to the DDM ($RT_{DDM}$) and a model parameter ($v_{RT}$) indicating to what extent the DDM-based *RT* influences the final received reward.

**1.10 Free parameters of the DDM**

This implementation of the combination of DDM and RML has several model parameters that can be seen as free parameters. These parameters are changed in our simulations to determine what model parameter values lead to the optimal estimation of the behavioural data by Vassena et al.(9) In this study, we did not change the parameters in the RML from Silvetti et al.(1), but only varied several of the indicated parameters that govern the DDM. These parameters are summarized in Table B in S1 Text. In total, four free parameters are estimated. The first parameter indicates to what extent the difference in expected value as estimated by the RML influences the DDM drift rate. A second parameter is used to indicate to what extent the boost is scaled to get the DDM threshold. A third parameter is used to determine the standard error of the Gaussian noise in the DDM. The final parameter is used to discount the RT output from the DDM, and convert this to a reward penalty.

| **DDM parameters** | | | |
| --- | --- | --- | --- |
| **Parameter** | Parameter description | Equation | Value used |
| $\boldsymbol{v}_{\boldsymbol{DDM}}$ | Scaling factor for the value difference in the DDM | S10 | 1.598 |
| $\boldsymbol{\theta}_{\boldsymbol{DDM}}$ | Scaling factor for the threshold in the DDM | S11 | 14.41 |
| $\boldsymbol{\sigma}_{\boldsymbol{DDM}}$ | Standard error of the Gaussian noise in the DDM | S12 | 71.99 |
| $\boldsymbol{v}_{\boldsymbol{RT}}$ | Scaling factor for the reaction time output | S13 | 62.04 |

***Table B****: Free parameters in the DDM external module for the speeded decision-making task. The table reports also the equations where each parameter is used and the optimal values from the behavioural data fitting.*

**1.11 Optimization of free parameters for the DDM**

In order to find the optimal values of the free parameters, we performed a gradient descent (GD) procedure on all four free parameters. The loss function of this GD procedure was a combination of the mean squared error of the accuracy and the response time outputs by the RML-DDM model compared to the real accuracy and response times reported by Vassena et al.(9) Here, accuracy is defined as the percentage of simulated trials the agent made the optimal decision (i.e. the decision leading to the highest reward). As the response time output by the DDM is unitless, and would only indicate the variable part of the response time (the non-decision time is not taken into account in our simulations), the RT was first z-scored before being added to the loss function. The parameter values corresponding to the optimal GD output are reported in the final column of Table B in S1 Text. The results of the behavioral data fitting for the RML-DDM and are shown in Fig A, panels A and B in S1 Text. Behavioral data from human participants were extracted from Vassena et al.(9) figures by means of WebPlotDigitizer software.


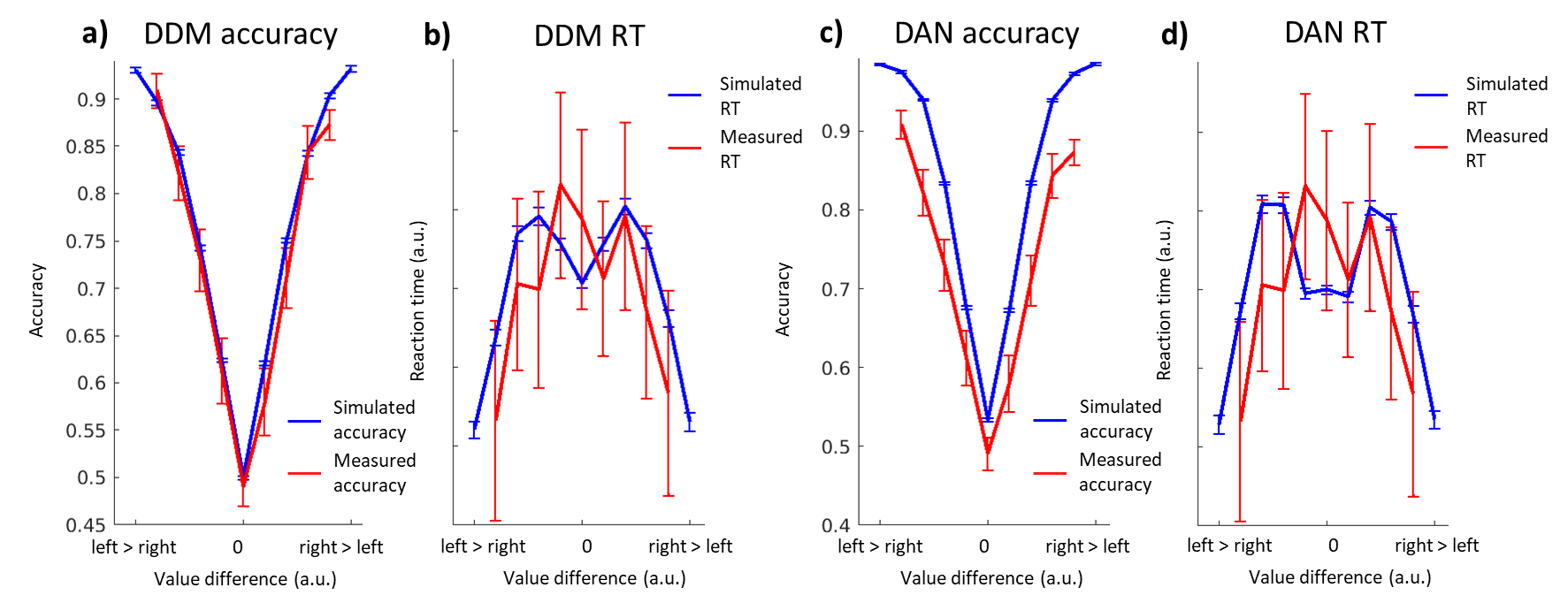


***Fig A: Results of the behavioural data fitting for the speeded decision-making task.*** *Simulated accuracy of the RML-DDM model at the optimal parameter values from Table B in S1 Text (blue line) and accuracy of the participants from Vassena et al.(9) (orange line).* ***b.*** *Comparison of optimal RTs simulated by the RML-DDM (blue line) and RTs from Vassena et al.(9) (orange line).* ***c.*** *Simulated accuracy of the RML-DAN model at the optimal parameter values from Table C in S1 Text (blue line) and accuracy of the participants from Vassena et al. (9) (orange line).* ***d.*** *Comparison of optimal RTs simulated by the RML-DAN (blue line) and RTs from Vassena et al. (9) (orange line).*

**1.12 The Dual attractor network**

In order to show the results attained using the RML-DDM combination are not solely due to the influence of the DDM, we implemented a second model. For this second model, we combined the RML with the dual attractor network (DAN), described by Usher & McClelland(11). Contrarily to the DDM, the DAN models decision-making not as a competition of one information variable, where two different boundaries determine which action is chosen, but using two different variables. These variables represent the information accumulation for both actions (i.e. the certainty the action is optimal). While these different variables (attractors) increase independently based on the information available in the environment, they are connected through mutual competition: each of these attractor states decreases the value of the other state proportional to its value. A decision is made when one of the two of these parameter values reaches a pre-set threshold.

This decision-making is modeled by accumulating information about both options over time. Each timestep, the information grows depending on the input, the perceived difference in information. Additionally, the attractors are considered to be leaky; with each timestep, the information decreases by a proportion of the current information. Finally, the attractors are competing. The information for one attractor state directly decreases the information for the other attractor state (see equations S14a and S14b, adapted from Usher & McClelland(11), with some changes in notation to avoid confusion with parameters from the RML).

|  | $x_{1}\left( t_{TR}+dt_{TR} \right)=x_{1}\left( t_{TR} \right)+dt_{TR}\cdot(I\left( 1 \right)-\kappa\cdot x_{1}\left( t_{TR} \right)-\beta_{DAN}\cdot x_{2}\left( t_{TR} \right)+\xi)$ | (S14a) |
| --- | --- | --- |
|  | $x_{2}\left( t_{TR}+dt_{TR} \right)=x_{2}\left( t_{TR} \right)+dt_{TR}\cdot(I\left( 2 \right)-\kappa\cdot x_{2}\left( t_{TR} \right)-\beta_{DAN}\cdot x_{1}\left( t_{TR} \right)+\xi)$ | (S14b) |

In this equation, ${x(t}_{TR})$ indicates the information for both options at the time, where ${x_{1}(t}_{TR})$ indicates the accumulated information for option 1, while ${x_{1}(t}_{TR})$ indicates the accumulated information for option 2. This information is updated using the information from the previous timestep, adding the input for options 1 and 2 (*I*(1) and *I*(2) respectively). From this value, part of the current value is subtracted (the decay of information, with a characteristic decay rate parameter $\kappa$). In this study, $\kappa$ is a model parameter, which is estimated during the parameter optimization step detailed below. Furthermore, the collected information for the other option influences the information for the current option; this information is multiplied with $\beta_{DAN}$ (renamed from the original equation by Usher & McClelland to avoid confusion with the $\beta$ parameter defined in the RML), a parameter indicating the strength of the inhibitory connection between the options, and subtracted from the information. Finally, random Gaussian noise ($\xi$) is added, with standard deviation *σ_DAN_*). In the simulations, $dt_{TR}$ is set to 1/10.

**1.13 RML-DAN interface**

In our current study, we combine the DAN with the RML. In this combination, several parameters from the RML are used as input for the DAN. These are used by the DAN to generate both a selected action and a reaction time of this choice, which are provided to the RML. Similar to the DDM implementation, the amount of cognitive control and the expected value of both actions are given as parameters to the DAN (see Fig B in S1 Text for a visual representation of the combination between the DAN and the RML). The amount of cognitive control influences the strength of the inhibitory connection between the options ($\beta_{DAN}$ in equations S14a and S14b), while the difference in expected value influences the difference in information gain between the actions (*I* in equations S14a and S14b).

The transformation of the level of cognitive control to the inhibitory connection strength is given in Equation S15: $\beta_{DAN}$ depends on the *NE_LC_*  value, multiplied with a scaling parameter ($Dual_{inf}$). Additionally, the difference in expected value between both options is used to determine the information gain parameter. The information gain parameter difference is set to the difference in value between options 1 and 2, multiplied with a scaling parameter ($Dual_{cong}$) (Equation S16a and S16b). As in Usher & McClelland(11), this value is added to 0.5 and subtracted from 0.5 in order to ensure a total information gain of 1.

|  | $\beta_{DAN}={NE}_{LC}/{NE}_{max}\cdot Dual_{inf}$ | (S15) |
| --- | --- | --- |

In this equation, *NE_LC_*  is from Equation S2, which is brought to a value between 0 and 1 by dividing it by its maximum theoretical value (which is 10).

|  | $I\left( 1 \right)=0.5+Dual_{cong}\cdot\left( v_{act}\left( 1 \right)-v_{act}(2) \right)$ | (S16a) |
| --- | --- | --- |
|  |  |  |
|  | $I\left( 2 \right)=0.5-Dual_{cong}\cdot\left( v_{act}\left( 1 \right)-v_{act}(2) \right)$ | (S16b) |

In equations 16 a and b, $v_{act}$(1) and $v_{act}$(2) represent the expected values of selecting options 1 and 2 according to the RML respectively.

The DAN then determines both the selected action and the value of $t_{TR}$ at the moment the action is selected and provides these values back to the RML. The value of $t_{TR}$ is then converted to the response time, and in the same way as with the RML-DDM combination, this response time is used to add a penalty to the received reward (see Equation S17). The slower the agent responds, the larger this penalty is.

| $R=RW_{act}-\frac{RT_{DAN}}{v_{RTDAN}}$ | (S17) |
| --- | --- |

In this equation, the trial reward (R) is a function of the received reward due to the chosen action ($RW_{act}$), and the penalty based on the response time. This penalty is found by dividing the found reaction time of this trial due to making a decision according to the DAN ($RT_{DAN}$) by a model parameter ($v_{RTDAN}$) indicating to what extent the DAN-based RT influences the final received reward.


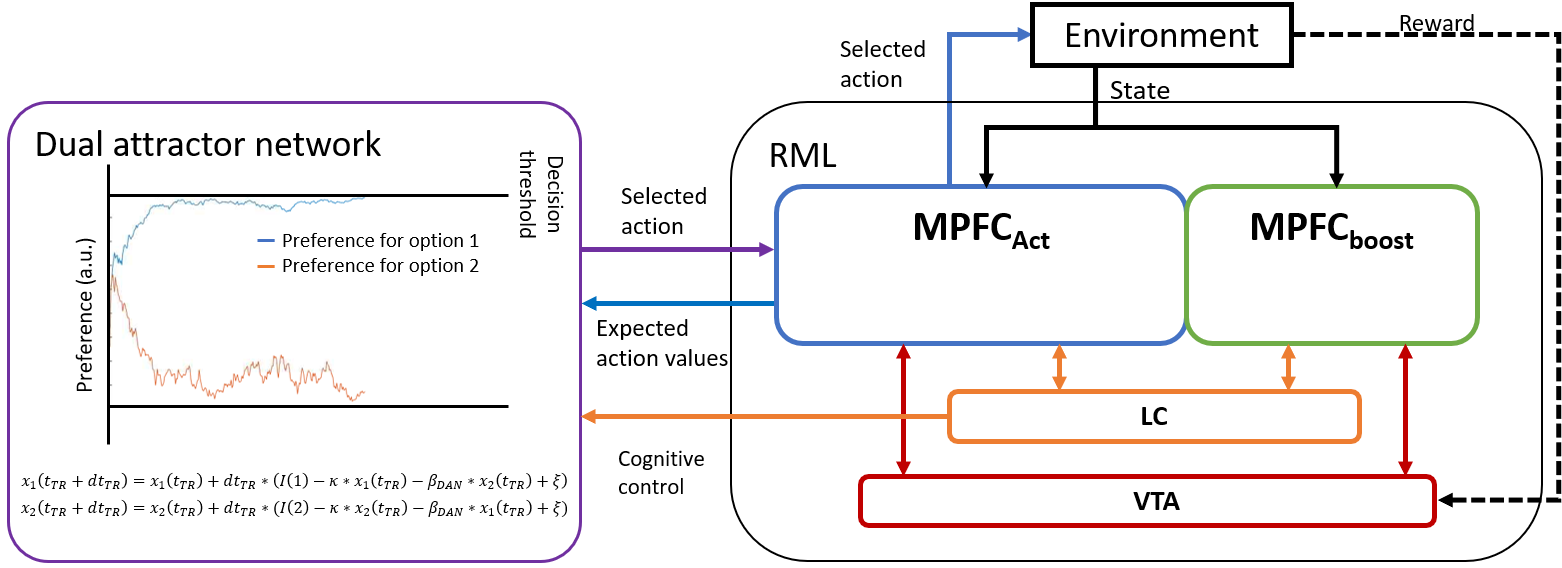


***Fig B****: Visual representation of the used connection between the RML and the DAN. As with the DDM representation, the DAN has a bidirectional connection to the action module of the dACC. On the one hand, the expected action values are taken from the dACC, and sent to the DAN. The difference between these action values is set as the difficulty of the trial. This value influences the external information gain (I) in each step in the dual attractor framework. This information gain is advantaged for the option with the higher expected reward. On the other hand, the DAN generates an action and response time, which are sent back to the action module of the dACC. There, they are combined with the received reward, and the RML updates the internal values for this action. Additionally, the DAN receives input from the LC. The amount of cognitive control determines the strength of the competition between both attractor states. A higher boost value would therefore allow an increase in information for one condition to inhibit the other condition stronger.*

**1.14 Free parameters of the DAN**

This implementation of the combination of DAN and RML has several model parameters that can be seen as free parameters. These parameters are changed in our simulations to determine what model parameter values lead to the optimal estimation of the data by Vassena et al.(9) As mentioned before, we did not change the parameters in the RML from Silvetti et al.(1), but only varied several of the indicated parameters that govern the combination of the DAN and the RML. These parameters are summarized in Table C in S1 Text. In total, five different free parameters are estimated. These are two free parameters in the update equations of the DAN (equations S14a and S14b), one for the characteristic decay rate, and one for the standard deviation of the Gaussian noise. Additionally, one scaling parameter is used for the value difference input, and one for the boost input. A fifth free parameter is used to convert the RT output from the DAN to a reward. These parameters are optimized (as described below), and the optimal values are shown in the final column of Table C in S1 Text. The comparison between human behavior and the simulated behavior is shown in Fig A, panels C and D in S1 Text.

| **DAN parameters** | | | |
| --- | --- | --- | --- |
| **Parameter** | Parameter description | Equation | Value used |
| $\boldsymbol{\kappa}$ | Characteristic decay rate | S14a, S14b | 0.408 |
| **σ_DA_** | Standard deviation of the Gaussian noise $\xi$ | S14a, S14b | 0.284 |
| $\boldsymbol{Dua}\boldsymbol{l}_{\boldsymbol{inf}}$ | Scaling factor for the information in the DAN | S15 | 0.727 |
| $\boldsymbol{Dua}\boldsymbol{l}_{\boldsymbol{cong}}$ | Scaling factor for the congruency in the DAN | S16a, S16b | 0.017 |
| $\boldsymbol{v}_{\boldsymbol{RTDAN}}$ | Scaling factor for the reaction time output | S17 | 99.63 |

***Table C****: Free parameters in the DAN external module for the speeded decision-making task. The table reports also the equations where each parameter is used and the optimal values from the behavioral data fitting.*

**1.15 Optimization of the DAN free parameters**

Similar to the DDM, the DAN free parameters are estimated using a GD procedure varying all five free parameters. As before, the loss function of this GD procedure was a combination of the mean squared error of the accuracy and the response time outputs by the RML-DAN model compared to the real accuracy and response times reported by Vassena et al.(9) As before, accuracy is defined as the percentage of simulated trials the agent made the optimal decision (i.e. the decision leading to the highest reward). As the response time output by the DAN is unitless, and would only indicate the variable part of the response time (the non-decision time is not taken into account in our simulations), the *RT* was first z-scored before being added to the loss function. The parameter values corresponding to the optimal GD output are reported in the final column of Table C in S1 Text. Behavioral data from human participants were extracted from Vassena et al.(9) figures by means of WebPlotDigitizer software.

**1.16 RML-C**

To show using the RML-C does not cause large changes in the results of the simulations, we additionally performed a simulation combining the RML-C with the DDM. The RML-C is described in detail by Silvetti et al. 2023(2). It is characterized by the addition of a curiosity term in the equation determining the VTA signals (see equations S4a and S4b), as the *tanh* of the expected surprise ($\hat{\delta}$), as defined in equation S8.

| $DA_{act,t}=(r_{t}\left( R_{t}+\mu b \right)+b\left( 1-\mu\right)\rho\max_{a\epsilon A_{s'}} (v_{act,t}(s',a)))-tanh(\hat{\delta})$ | (S18a) |
| --- | --- |
| $DA_{boost,t}=r_{t}R_{t}-\omega b+\max_{b\epsilon B_{s'}} (v_{boost,t}(s',b)))-tanh(\hat{\delta})$ | (S18b) |

For the RML-C simulations, we used the DDM optimal parameters from Table B in S1 Text.

**1.17 Verbal working memory task modeling**

During each trial (Fig 4A, main text), 1, 4, 6 or 8 words were presented to the model, generating four different difficulty levels. After a delay of 10s, the model was presented with a target word that matched one of the memorized words in 50% of trials. The model’s goal was to indicate whether the target word matched one of the words presented before. In case of correct response, the model received a reward signal equal to 3, while it received no reward for an incorrect response. The RML first performed a training session, consisting of 40 trials for each difficulty level (160 trials). Afterwards, it performed the task, consisting of 90 trials for each difficulty level (360 trials in total), randomly intermixed. We repeated the simulation 20 times (simulating 20 participants). RML parameters were the same as in the original paper (1) (Table A in S1 Text).

**1.18 Working memory (WM) model**

The RML was connected to a task-specific external module (Fig 4B, main text) simulating items encoding and maintenance in the WM (12). This module consisted of a two-layered competitive recurrent neural network (cRNN). The input layer (16 units) encoded the words (both target and words to be memorized), while the output layer (16 units) maintained the words, thanks to recurrent connectivity with the input layer. We assigned arbitrarily one input unit to each word. Each output unit was connected with symmetrical weights to one input unit. Output units were globally connected with symmetric lateral inhibitory weights. We arbitrarily assigned a duration of 10 ms for each network update cycle. For this reason, for example, a delay of 10s meant 1000 network cycles. The lateral inhibitory connections in the output layer ensured an overall decrease of activity as a function of the number of active units, simulating the detrimental effect of increasing WM load on item retention(12). Equations and a more detailed description of the cRNN, including the parameters set we used in this study, can be found in the Supplementary Material of our previous study(1).

**1.19 RML-cRNN interface**

The RML-cRNN interface from our previous study was designed for a dynamical implementation of the RML(1). Here, we redesigned the interface, as in this study we used an MDP implementation of the RML. The activity of the cRNN output layer was modulated by the NE signal from the LC module of the RML (Equation S2), defining a variable *h* indicating a preference for selecting the ‘match’ action, based on the activity of each neuron in the output layer of the cRNN (Equation S19):

| $h={NE}_{LC}\cdot\max\left( F \right)-\epsilon$ | (S19) |
| --- | --- |

Where *F* is the vector of the cRNN output layer activity, *h* is a scalar resulting from the activity modulated by the LC output *NE_LC_* subtracted by a threshold parameter $\epsilon$ *= 0.15.* When *h* > 0, the RML has more evidence for a match trial, while a value of h < 0 indicates that the RML has more evidence for a mismatch trial. For each trial, action selection about the presence of a match is performed by Equation S3, selecting between the ‘match’ action (indicating a match is present), and the ‘mismatch’ action (indicating a match is absent), via modulation of the state-action values as stated in equations S20a and S20b:

| $v_{act}^{*}\left( s,a_{match} \right)=v_{act}\left( s,a_{match} \right)+\varphi\cdot h$ | (S20a) |
| --- | --- |
|  |  |
| $v_{act}^{*}\left( s,a_{mismatch} \right)=v_{act}\left( s,a_{mismatch} \right)- \varphi\cdot h$ | (S20b) |

Where $v_{act}^{*}$ is the cRNN-modulated state-action value (to be used in Equation S3), $a_{match}$ indicates the index of the ‘match’ choice, $a_{mismatch}$ indicates the index of the ‘mismatch’ choice, $\varphi=50$ is a scaling parameter, and $h$ is the preference variable, from Equation S19.

**1.20 Foraging decision task modeling**

In a third simulation, we modeled a task where the RML decided between foraging options and engage options (Fig 5A and Methods in the main text). We designed the task on the basis of the foraging tasks proposed in earlier literature studies investigating the effects of the dACC in foraging (13,14). During each trial, the RML is shown a compound cue containing information about the current context (patch), and the reward available in the current trial. In response to this cue, the RML can opt to forage, in which the current cue indicating the reward available is replaced by a different cue available in the context. In this case, the RML views a new compound cue, consisting of the same context, and the newly selected reward cue, and is given the same decision to either forage or engage. In case the model decides to engage, it is presented with a two-armed bandit task, where they can obtain a reward indicated by the reward cue. This two-armed bandit consisted in deciding between two options with a fixed value. One of these options yields a reward equal to the reward indicated by the cue, while the other yields a reward that is at most .5 different from the reward indicated by the cue. We simulated 16 contexts, each generating 8 different bandit games. The context values were determined by the average value of the bandit games they generated (foraging values were set between 2.86 and 6.14). Each trial continued until the RML decided to engage, and performed the subsequent two-armed bandit task, or the RML decided to forage for 20 times consecutively. The model received a reward only after having played a bandit game. Each forage option was followed by a state where the RML passively viewed the cue of the current context, before transiting back to the initial state where another bandit game (randomly sampled from the current context) was proposed to the model. Each trial had a timeout for the response time (2000 DDM cycles), after which the trial was aborted and no reward was delivered. We administered 36 trials for each bandit game of each context, for a total of 4608 trials. Before the task execution, the RML was extensively trained to link the cues to the respective values of the contexts and of the bandit games. Finally, neural data analysis was conducted on both boost and whole dACC activity. The latter was computed as the sum of the normalized (divisive normalization) mean boost and mean value. In total, 40 simulations were performed, and the average results were reported.

**1.21 Combination of the RML with the DDM**

Like for the speeded decision-making, here we connected the RML to a DDM to perform the task, in order to simulate both decision-making and RTs. The RML-DDM interface was similar to the speeded decision-making task, with only one difference of the introduction of a bias term that could be modulated by the LC output. Human participants have indeed a bias to select the engage option(14). To reflect this, we added a bias term (*bias*) to the RML, which promoted by default the “engage” option. This bias toward engaging was discounted by the LC output as indicated in Equation S21, so that the higher the control signal, the lower the bias for engaging (therefore the higher the probability of choosing to forage):

| $bias=bias_{DDM}/NE_{LC}$ | (S21) |
| --- | --- |

For this task, we did not implement a time pressure factor, therefore the reward signal was not discounted by the RT. In the simulations, *dt* was set to 1, and a cutoff of 2000 time steps was used, after which the trial was aborted and no reward was delivered.

**1.22 Optimization of the foraging decision task**

The DDM parameters were optimized using a gradient descent procedure, aiming to minimize the difference between the reported behavior in Shenhav et al.(14) and the simulated behavior. The loss function used was the sum of the mean square error of the foraging probability, and the mean square error of the z-scored, log-transformed reaction time (as they are reported in the original work). The parameters of interest are the same parameters optimized in the speeded decision-making task, with the exception of $v_{RT}$ (not present in this simulation). Instead, the parameter governing the bias (see Equation S21) was optimized. The optimal parameters are summarized in Table D in S1 Text. The comparison between the simulated behavior and the behavior reported by Shenhav et al.(14) is shown in Fig C in S1 Text. Behavioral data from human participants were extracted from Shenhav et al.(14) figures by means of WebPlotDigitizer software.

| **DDM parameters** | | | |
| --- | --- | --- | --- |
| **Parameter** | Parameter description | Equation | Value used |
| $\boldsymbol{v}_{\boldsymbol{DDM}}$ | Scaling factor for the value difference in the DDM | S10 | 1.519 |
| $\boldsymbol{\theta}_{\boldsymbol{DDM}}$ | Scaling factor for the threshold in the DDM | S11 | 12.41 |
| $\boldsymbol{\sigma}_{\boldsymbol{DDM}}$ | Standard error of the Gaussian noise in the DDM | S12 | 3.014 |
| $\boldsymbol{bias}_{\boldsymbol{DDM}}$ | Bias towards the engage option | S21 | 22.55 |

***Table D:*** *Free parameters in the DDM external module for foraging task. The table repots also the equations where each parameter is used and the optimal values from the behavioral data fitting.*


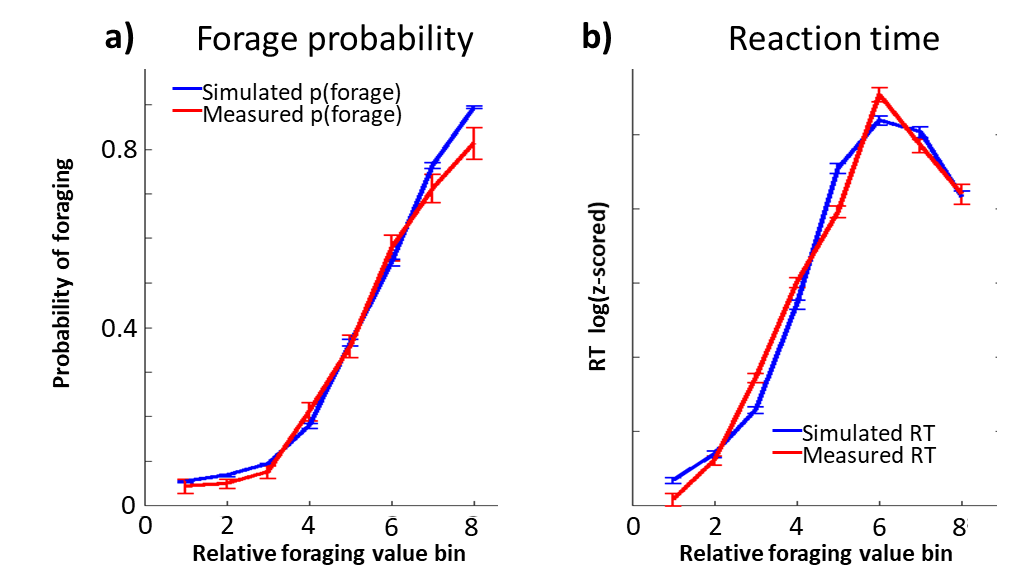


***Fig C. Behavioural data fitting results for the foraging task.*** ***a.*** *Simulated probability of foraging of the RML-DDM model at the optimal parameter values from Table D in S1 Text (blue line), and of the participants from (14) (red line). The plot shows the probability of foraging in eight different bins depending on the relative foraging value (i.e. the difference between the foraging value and the engage value) of the trial. Smaller bin number indicates smaller relative foraging value.* ***b.*** *Same as in a, but about RTs. The plot shows the mean of the log transformed, z-scored RTs.*

**2 Supplementary results**

**2.1 Behavioural data fitting**

In order to predict the neural data, we first optimized the parameters of the task-dependent modules to fit the behavioural data from the original fMRI studies. Fig A and Fig C in S1 Text show the simulated behavioural data at the optimal points of parameters space.

**2.2 Speeded decision-making task with RML-DAN combined model**

The results from the RML-DAN combined model are shown in Fig D in S1 Text. In Fig D, panel A in S1 Text, we have shown the dACC activity as measured by Vassena et al.(9), in order to compare this activity to the simulated dACC activity by the RML-DAN combined model (shown in Fig D, panel B in S1 Text). Similar to the results of the RML-DDM combined model, we can see that the simulated dACC activity follows a W-shaped pattern (the dashed blue line in Fig D, panel B in S1 Text), closely reproducing the found dACC pattern. After using the AIC to test the most likely order of the simulated dACC activity (as described by Wagenmakers & Farell(15)), we find that the simulated dACC activity is more likely to be a quartic function of the value difference rather than a quadratic one with a positive leading coefficient (Akaike weight > 0.999, equivalent to a p-value < 0.001). This dACC activity is the sum of the mean expected value (Fig D, panel C in S1 Text), and mean cognitive control (Fig D, panel D in S1 Text). The mean expected value shows (Fig D, panel C in S1 Text), like in the RML-DDM combined model, a u-shaped function. We can see an increase in expected value with a larger value difference. The minimum in the expected value when the value difference is close to 0 can be explained in two ways. First, when the value difference is small, the difference in information gain is small (see Equation S15). This leads to a situation where the accumulated information for both options is equal. Since the accumulated information for one option inhibits the information for the other option (see Equations S14a and S14b), this causes the *RT* to increase, in turn causing a larger penalty term (see Equation S17), and thus a lower value. Secondly, a higher value of boosting causes a larger cost due to the intrinsic cost of boosting. Since a lower value difference increases the boost value (as detailed below), these trials yield a lower value compared to the trials where there is a large difference in value. The mean cognitive control function (Fig D, panel D in S1 Text) shows a similar shape to the mean cognitive control function: an inverted U-shaped function. Since boost carries an intrinsic cost, the boost is only increased when necessary, yielding an increase in boost when the trial is difficult (i.e. when the difference between the options is small). In these cases, an increase in boost level lowers the inhibition between the two accumulators, allowing for a faster *RT*. This has two advantages: first, it directly reduces the penalty term to the reward. Second, it allows the RML-DAN combined model to respond in time in relatively more trials.


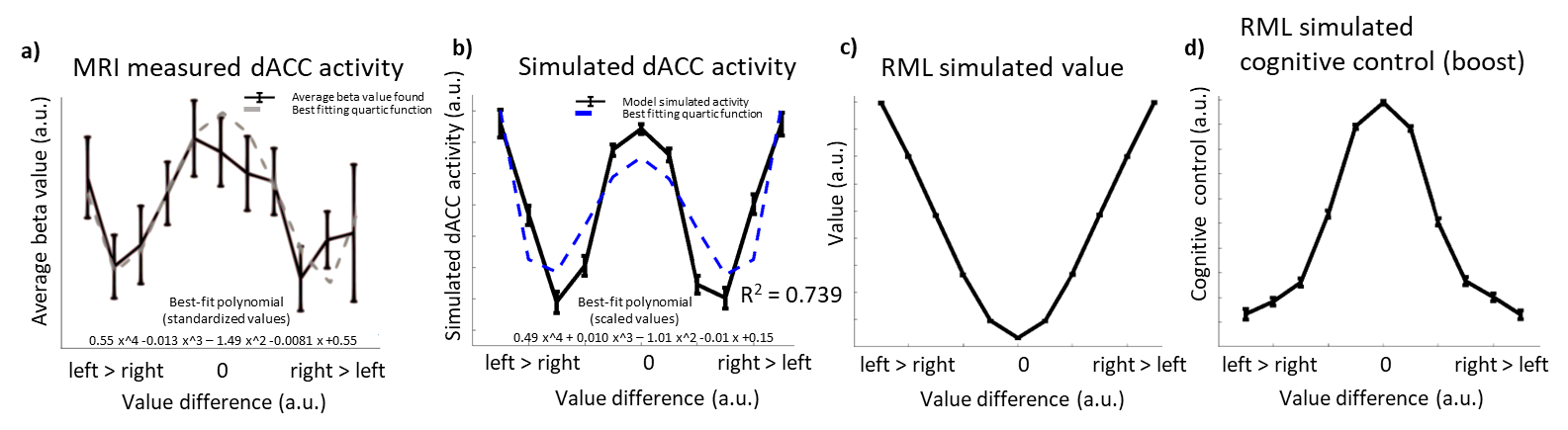


***Fig D****: Results from the DAN combined with the RML.* ***a****: The real dACC activity measured using fMRI by Vassena et al.*(9) ***b:*** *Solid line: the dACC activity as simulated using the RML combined with the DAN. This activity is a sum of the expected value and cognitive control simulated by the RML. The dashed line is the best-fitting quadratic function. We can see that similar to the fMRI data, the quartic function fitting the simulated dACC activity has a positive first coefficient.* ***c:*** *The expected value simulated for different value differences. This expected value shows a u-shaped function, with a maximum for the extreme value differences.* ***d:*** *The simulated cognitive control level for different value differences. Contrary to the value function, the cognitive control shows an inverted u-shaped function, with a maximum value when the value difference is 0.*

**2.3 RML-C results**

The results from the RML-C combined with the DDM are shown in Fig E in S1 Text. Fig E, panel A in S1 Text shows the simulated dACC activity when using the RML-C rather than the RML combined with the DDM. This activity is the sum of the RML simulated value (plotted in Fig E, panel B in S1 Text) and the RML simulated cognitive control (plotted in Fig E, panel C in S1 Text). As expected, the simulated data do not differ much from the RML-DDM data. Importantly, when calculating the AIC(15), the simulated dACC activity is still a quartic function rather than a quadratic function of the value difference (Aikake weight for a quartic function > 0.999).


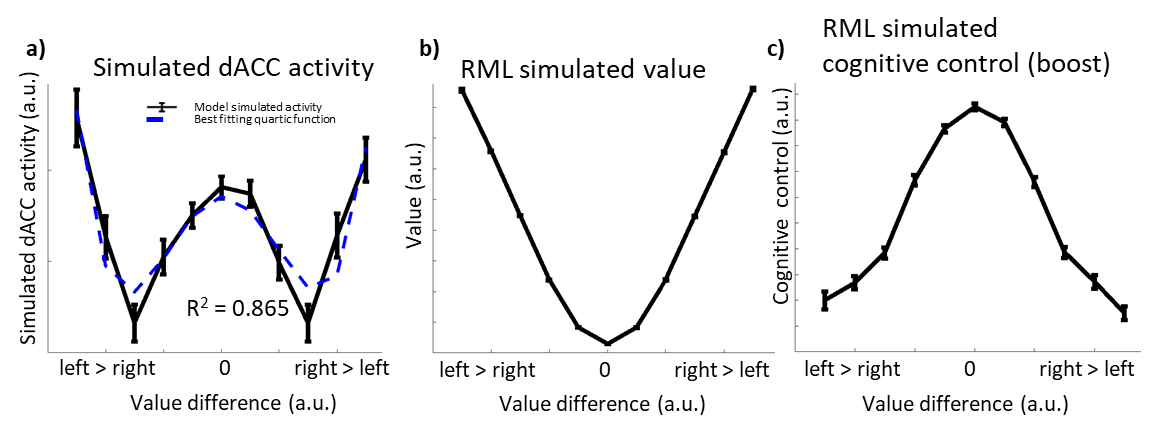


***Fig E****:* ***RML-C results****.* ***a****: The mean dACC activity for each of the 11 tested differences in value as simulated by the RML-C (black line), and the best fitting quartic function (blue, dashed line). This activity is the sum of the value (panel b) and the cognitive control (panel c).* ***b****: The value component of the RML-C-simulated activity.* ***c****: The cognitive control simulated by the RML-C.*

**2.4 Effect of changes in the *v_RT_* and *v_RTDAN_* parameter on the simulated dACC activity in the speeded decision-making task**

Besides a change in the parameters governing the interface between the RML and the DDM or DAN, one of the parameters we base our model inversion on is the parameter governing how the amount of steps the model takes is converted into a reward devaluation for the model. In order to investigate the stability of the dACC simulations with changes in this parameter, we have performed the simulation for several different values of either *v_RT_* (in case of the DDM) or *v_RTDAN_* (in case of the DAN), changing the value from the optimal by a certain percentage between an addition of 75%, and a subtraction of 75%. Fig F in S1 Text shows the effects of a change in this parameter. The RML-DDM combined model shows mild effects on the simulated dACC activity after a change in the *v_RT_* parameter. The RML-DAN combined model does show some differences in dACC activity when changing the *v_RTDAN_* parameter. Although an increase does not affect the shape of the simulated dACC activity function too much, a large decrease (75%) shows a change from a quartic function to a sixth-degree polynomial function.


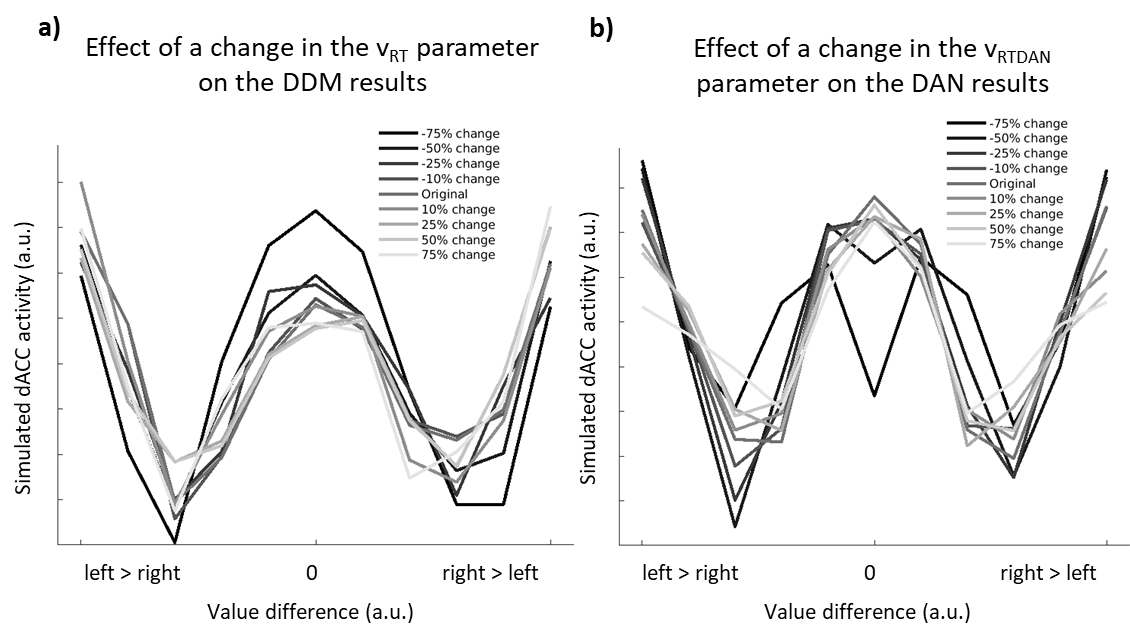


***Fig F****:* ***Effect of different time pressure levels on the simulated dACC activity.*** ***a)*** *Simulated dACC activity is mildly influenced by different levels of time pressure to respond (v_RT_ parameter in Equation S13). Original: v_RT_ value used in the simulation shown in the main text (Table B in S1 Text). Variations as a percentage of the original value. Results from the RML-DDM simulations.* ***b)*** *Same as in a), but relative to the RML-DAN simulations. Original: v_RTDAN_ value reported in Table B in S1 Text and used in Equation S17.*

**References**

1. Silvetti M, Vassena E, Abrahamse E, Verguts T. Dorsal anterior cingulate-brainstem ensemble as a reinforcement meta-learner. PLOS Computational Biology. 2018;14(8):e1006370. doi: https://doi.org/10.1371/journal.pcbi.1006370

2. Silvetti M, Lasaponara S, Daddaoua N, Horan M, Gottlieb J. A Reinforcement Meta-Learning framework of executive function and information demand. Neural Networks. 2023;157:103–13. doi: https://doi.org/10.1016/j.neunet.2022.10.004

3. Kalman RE. A new approach to linear filtering and prediction problems. Transactions of the ASME - Journal of Basic Engineering. 1960;82:35–45. doi: https://doi.org/10.1115/1.3662552

4. Doya K. Metalearning and neuromodulation. Neural Networks: The Official Journal of the International Neural Network Society. 2002;15(4–6):495–506. doi: https://doi.org/10.1016/s0893-6080(02)00044-8

5. Sara SJ. The locus coeruleus and noradrenergic modulation of cognition. Nature Reviews Neuroscience. 2009;10(3):211–23. doi: https://doi.org/10.1038/nrn2573

6. Sara SJ, Bouret S. Orienting and reorienting: the locus coeruleus mediates cognition through arousal. Neuron. 2012;76(1):130–41. doi: https://doi.org/10.1016/j.neuron.2012.09.011

7. Köhler S, Bär KJ, Wagner G. Differential involvement of brainstem noradrenergic and midbrain dopaminergic nuclei in cognitive control. Human Brain Mapping. 2016;37(6):2305–18. doi: https://doi.org/10.1002/hbm.23173

8. Silvetti M, Seurinck R, van Bochove ME, Verguts T. The influence of the noradrenergic system on optimal control of neural plasticity. Frontiers in Behavioral Neuroscience. 2013;7:160. doi: https://doi.org/10.3389/fnbeh.2013.00160

9. Vassena E, Deraeve J, Alexander WH. Surprise, value and control in anterior cingulate cortex during speeded decision-making. Nature Human Behaviour. 2020;4(4):412–22. doi: https://doi.org/10.1038/s41562-019-0801-5

10. Ratcliff R. A theory of memory retrieval. Psychological Review. 1978;85(2):59–108. doi: https://doi.org/10.1037/0033-295X.85.2.59

11. Usher M, McClelland JL. The time course of perceptual choice: the leaky, competing accumulator model. Psychological Review. 2001;108(3):550–92. doi: https://doi.org/10.1037/0033-295x.108.3.550

12. Ashby FG, Ell SW, Valentin VV, Casale MB. FROST: a distributed neurocomputational model of working memory maintenance. Journal of Cognitive Neuroscience. 2005;17(11):1728–43. doi: https://doi.org/10.1162/089892905774589271

13. Kolling N, Behrens TE, Mars RB, Rushworth MF. Neural mechanisms of foraging. Science (New York, NY). 2012;336(6077):95–8. doi: https://doi.org/10.1126/science.1216930

14. Shenhav A, Straccia MA, Cohen JD, Botvinick MM. Anterior cingulate engagement in a foraging context reflects choice difficulty, not foraging value. Nature neuroscience. 2014;17(9):1249–54. doi: https://doi.org/10.1038/nn.3771

15. Wagenmakers EJ, Farrell S. AIC model selection using Akaike weights. Psychonomic Bulletin & Review. 2004;11(1):192–6. doi: https://doi.org/10.3758/BF03206482
