# Supplementary material for: Meta-Reinforcement Learning reconciles surprise, value, and control in the anterior cingulate cortex
Source: PLoS Comput Biol. 2025 Apr 22;21(4):e1013025. doi: 10.1371/journal.pcbi.1013025 (PMC12047761; doi:10.1371/journal.pcbi.1013025)
Supplement: Attachment — Submitted filename: Response to Reviewers.pdf [file pcbi.1013025.s003.pdf]

Response to reviewer's comments.

The reviewers can find below the point-to-point responses to their comments. Based on the reviewers' request and suggestion, the manuscript underwent three macroscopic changes. First, following the requests of Reviewer #1, we implemented a third simulation related to the foraging value theory. Second, following the suggestion of Reviewer #3, we deeply revised the manuscript structure, organizing it into four macro-sections: Introduction, Methods, Results, and Discussion. Third, based on the comments by Reviewer #2, the Discussion section was extended, including a paragraph on limitations and future directions. Besides these three macro-revisions, we took care of improving the manuscript following all the reviewer's suggestions.

Finally, while analyzing the RML simulations of response times (based on the request of Reviewer #1), we realized that, in the simulation relative to the speeded decision-making task, the optimization of the DDM parameters prematurely stopped before reaching a minimum. For this reason, we re-ran the optimization and the corresponding simulation, finding equivalent results. However, due to the new simulation, the reviewers can find slightly different plots in the figure relative to the speeded decision-making task (new Figure 3).

Along with this response to the reviewer's comments, we have attached the revised manuscript, and a version of the revised manuscript where the changes have been highlighted as blue text (which can be found at the end of this document). Similarly, we have included two versions of the supplementary methods: the revised supplementary methods and a version of the revised supplementary methods where the changes have been highlighted as blue text.

The authors want to express their gratitude for the high quality of the reviews, which led to a noticeable improvement in the manuscript clarity and impact.

In their study titled "Meta-Reinforcement Learning reconciles surprise, value, and control in the anterior cingulate cortex.", Vriens et al. compare the predictions of meta-reinforcement learning (MRL) model simulations to actual signals recorded in the dorsal anterior cingulate cortex (dACC). The topic is timely, as several incompatible theories exist, and a consensus is still lacking (see Clairis & Lopez-Persem, 2023 for a full explanation of these theoretical conflicts). Therefore, studies like this one are necessary in the field of cognitive neuroscience.
In their article, the authors found that predictions from their model, which had previously been introduced in several publications, matched the dACC signals recorded during two tasks: a speeded binary choice task and a verbal working memory task. While the methods are robust and the findings are interesting, I believe that more in-depth analysis is required to fully support their main claim—that they resolve the theoretical impasse surrounding the function of the dACC (this claim should be alleviated). Some relevant literature, particularly studies that conflict with their framework, is not covered sufficiently. Despite these weaknesses, I believe the manuscript can be significantly improved with major revisions.

We would like to thank the reviewer for the assessment and comments regarding our manuscript. Following the Reviewer's suggestions, we implemented a series of in depth revisions described below. We also alleviated the claim of a final resolution of the theoretical impasse.

Theoretical aspects of the manuscript (introduction and discussion):
1. In the abstract and introduction, the authors state that two competing frameworks, the EVC and the performance monitoring framework, attempt to find a unified theory of dACC function. However, other studies suggest that the dACC function is also related to foraging value (see Clairis and Lopez-Persem 2023 for an overview of these theories). To be more inclusive, I recommend that the authors briefly integrate this literature (Kolling and colleagues work) into both the introduction and discussion.

We are grateful for the reviewer pointing out this missing part of the literature and agree with the necessity of adding this line of studies to the paper. To improve the document, we integrated this work into the Introduction and Discussion of the manuscript. Moreover, we extended our work by administering to the RML also a foraging task.

2. In the introduction, the authors state: "Furthermore, a recent fMRI study compared the predictions of the two frameworks during a speeded decision-making task, which involved both cognitive control and performance monitoring32." Indeed, but this was challenged by Shenhav A, Musslick S, Botvinick MM, Cohen JD. Misdirected vigor: Differentiating the control of value from the value of control. PsyArXiv. [Preprint]. 2020. doi:10.31234/osf.io/5bhwe. Could the authors address the criticisms raised in this commentary and explain how they dissociate control from vigor in their manuscript?

While this comment opens up an interesting debate, we would first like to point the reviewer to a recent response to this preprint by Vassena et al. (https://doi.org/10.31234/osf.io/ugjrf), where the authors show that the mentioned preprint by Shenhav et al. misinterpreted the different models simulated in their original paper. We agree that pointing out that there is an open debate on this topic is important. For this reason, we have added a short section on this debate in the new "Limitations" paragraph in the Discussion, where we also stress that the current theoretical impasse would hold regardless of the results from the speeded decision-making task. This is because it raises from the complementarity of the performance monitoring and the cognitive control frameworks, where the former can account for a large set of experimental results evidencing the dACC role in surprise coding (e.g. see Alexander & Brown 2011; Silvetti et al., 2011; Silvetti et al., 2014), while the latter can account an equally large set of findings showing the dACC role in effort (cognitive or physical) coding (e.g. Vassena et al., 2014; Engstrom et al., Engstrom et al., 2009).

3. The article from Silvetti et al, 2018 (ref 34 in the manuscript) already attempts to demonstrate that the RML is efficient in explaining the dACC function. Can the authors clarify the novelty of their findings in comparison to previous work?

Here we extend the 2018 RML findings to three new experimental paradigms: a speeded decision-making task, a verbal working memory task, and a foraging task. The novelty consists of the direct comparison between the predictions of the RML and the other two frameworks right in the experimental paradigms that are central to the current theoretical crisis. In particular, the RML simulations relative to the speeded decision-making and the foraging tasks were not intuitively derivable from the previous paper. Finally, for what concerns cognitive effort in the WM task (simulated also in Silvetti et al., 2018), in this work we paid particular attention to avoid any confound related to surprise and we administered to the RML an experimental paradigm closer to the one used by Engstrom et al.. We clarified these points in the Introduction section.

4. From the discussion, I understand that RML is not the most recent model, so why not use RML-C?

The RML-C differs from the RLM in its utility function, which includes a term coding for uncertainty. This leads the RML-C to both maximize the net value (extrinsic motivation, like the RML) and minimize uncertainty (intrinsic motivation; Silvetti et al. 2023). The tasks we used in this work were dominated by the extrinsic motivation component, for this reason, we parsimoniously opted for the RML (see revised Discussion). Moreover, the RML and the RML-C predictions in tasks not involving

intrinsic motivation are very similar (Silvetti et al., 2023), as we now show in the Supplementary Results, where we replicated the simulations of the speeded decision-making task with the RML-C.

On the choice of experimental designs:
5. The article would be much more convincing if simulations were conducted on additional experimental designs that are known to trigger dACC activity. For instance:
• Why simulate a simplified version of the choice task from Vassena et al.? What are the results of the actual task from Vassena et al.?

We acknowledge that declaring in our manuscript a simplification of the original task without any further clarification can raise some doubts in the reader. The simplification we operated on the original task by Vassena et al. consisted of administering, in each trial, a two-armed bandit where the agent was asked to choose between two stimuli. Differently, in the original paper, the visual pattern for humans was more complex as they were asked to choose between two pairs of stimuli, therefore the value of each option was a combination of the values of the two figures making a pair. Our simplification was aimed at reducing the implementation complexity of the simulations, without changing the original task structure. We modified the task description, providing some clarifications about our choice. Moreover, in the revised version of the manuscript, we added a third simulation to investigate the RML activity during a foraging task, including in this way an additional experimental design in our work.

• How does the model perform on tasks designed by Kolling et al., which show that the dACC encodes foraging value?

As anticipated above, we included a third simulation where we tested the RML predictions in a foraging task (Kolling et al. 2012; Shanhav et al., 2014; Brown & Alexander, 2017). We thank the reviewer, as we acknowledge that this third section of the manuscript notably improved the width of our work.

• What are the model's predictions for tasks involving effort (such as those in Clairis and Pessiglione 2022, 2024)?

The RML predicts that effort (both cognitive and physical) is optimized to maximize the net value, i.e. the performance improvement discounted by the intrinsic cost of effort exertion (Silvetti et al., 2018, 2023; see also Silvestrini et al. 2023 for an overview). Besides citing the results from our previous works, in the current work, we administered a cognitive effort task to the RML (verbal WM task), where effort was not confounded by surprise.

We thank the reviewer for making us aware of the works by Claris and Pessiglione, which are relevant to our work. Both these works have shown an MPFC functional differentiation, with vm areas coding for value and dorsal areas coding for effort, in agreement with our results from the speeded decision-making task simulation (Figure 3 in the revised manuscript). We agree that this point might be unclear to the reader in the current manuscript, and decided to introduce this relevant literature in the Discussion section.

Results:
6. One of the most consistent findings in binary choice tasks is that the dACC encodes the value of the unchosen option positively and the value of the chosen option negatively. Consequently, the decision value (Chosen-Unchosen) is coded negatively. In this paper, the authors show results from a previous fMRI experiment and simulations depicting a value difference in the side frame (i.e.,

right vs. left option values). It seems that what they call a quartic relationship might actually correspond to a quadratic relationship in terms of decision value (DV, choice frame) with the dACC signal. I have several questions and suggestions in this regard:

o Can the authors justify their choice of the side frame?

The authors hope they have been able to understand correctly the reviewer's comment. The frame about options value was balanced by side. This means that the agent made its decisions only based on the value of the different options and the level of cognitive control implemented, regardless of the side of the best option, which was pseudo-randomly assigned. This explains the symmetry of our results with respect to the side. This point is now better specified in the task description, Methods section.

o To my knowledge, only one article demonstrates this quartic relationship. Many datasets with binary choice tasks are available on open platforms. It would be reassuring if the authors could demonstrate that this quartic relationship holds in other datasets.

Although several studies adopting versions of the 2-armed bandit and speeded decision-making tasks can be found in the cognitive neuroscience literature, the novelty of Vassena's et al. work consisted also in the specificity of the task they used. Indeed, their task was designed ad hoc to manipulate surprise, cognitive control, and value to compare specifically PRO and EVC predictions. We acknowledge that a replication of Vassena's et al. fMRI results is desirable and we included this point as a limitation in the Discussion section.

o How sensitive is this quartic relationship to time pressure? What are the model's predictions without time pressure?

The Reviewer can find the analysis of the effect of time pressure variability on neural predictions in Supplementary Figure 5, where we show that the W-shaped neural response is consistent across different levels of time pressure.

Based on our task representation in the DDM module, without any time pressure there would be no need for cognitive control: the agent would have infinite time to correctly integrate the evidence. In that case, the optimal boost level would be always the minimal one, to minimize the cost associated with boosting.

From an ecological viewpoint, we expect time pressure to be present to some extent in many different tasks, also when that is not explicitly communicated in the task instructions, by virtue of opportunity cost.

o What are the model's predictions regarding the encoding of chosen and unchosen option values?

The RML implementation that we used in this work inherits from Q-learning the encoding of state-action values in Q matrices (in Silvetti et al. 2018, a continuous time neural network-based version of the model was implemented as well). For this reason, both chosen and unchosen option values are represented in the Q matrices, but like in classical Q-learning algorithms, only the Q-value of the chosen option is updated, thanks to action-outcome comparison.

o A low DV can correspond to high-high or low-low options. Does the model make different predictions for these two cases? One might expect that high-high options require less "effort," as the consequences of both actions are positive.

The RML predicts a control intensity (boost) for high-high choices greater than or equal to the one for low-low choices. This is due to three reasons. 1. We assume the decision is made as an evidence accumulation process (DDM or DAN), therefore a smaller value difference leads to a longer RTs. This means that both high-high and low-low trials come on average with longer RTs. 2. We assume there is a time pressure factor, so that the slower the decision, the smaller the reward (in case of timeout, the reward can be even absent). This means that the value of both high-high and low-low trials is strongly discounted due to long RTs. 3. Cognitive control (boost) can speed up the evidence accumulation process, leading to a shorter RT and then to a higher reward. However, boost comes with a cost, therefore the intensity of the control signal is optimized as a function of the expected reward.

For these three reasons, the RML maximizes boost (then it minimizes the RT) in the high-high trials, where mitigating the detrimental effect of time results in large reward (high cost of control but also high expected gain). In the case of low-low trials, shortening the RT would lead to a small reward anyway, justifying only a mild increment of boosting, due to the cost-benefit trade-off.
In the revised version of the manuscript, we provided a qualitative explanation of the boost management in the RML in the Results subsection dedicated to the speeded decision-making task.

o What are the model's predictions regarding response times?

We have added the figures for the predicted response times to the Supplementary Results (supplementary figures 1,3). For both the speeded decision-making task and the foraging task, the RML provided a good fit for the human data.

7. Regarding the anatomical definition of the dACC: the brain regions involved in the speeded decision-making task and the verbal WM tasks are not the same. One difficulty in establishing a consensus on dACC function is to clearly delineate which brain region is being studied. Is the signal in the dACC, as defined by the ROI in the WM task, similar to the one identified in the speeded decision-making task? Is the reverse also true?

To clarify this point, we have added Figure 6, showing the overlap between the dACC clusters from all the fRMI studies we analyzed.

Minors:
Terminology: Rather than stating that the MRL "can explain" the dACC function, it would be more accurate to say that it "can mimic" or "resembles" the dACC function, as the results are based on simulations.

This is an interesting epistemological problem. We reduced the frequency of the verb "to explain" in favor of "to predict", although we would like to stress that models like PRO or the RML are mechanistic models, not statistical, data-driven, models.

Reviewer 2:

Response to reviewer 2

Reviewer #2: This manuscript presents the results of two simulations using a reinforcement meta-learner (RML) model to model the activity of the dorsal anterior cingulate cortex and compare it with published fMRI results. The aim is to test the hypothesis that this brain region integrates outcome/performance/surprise monitoring and cognitive control. While this is not a novel hypothesis, the authors are correct in stating that previously proposed mathematically formalized models are usually capable to explain only one of these two aspects. The RML model is applied to a speeded decision making task and a working memory task and captures the neuroimaging results by predicting both cognitive control and monitoring functions.

This is an interesting and well-written manuscript. The methods are sound and well-explained in the supplementary materials. I have only very few and minor comments that may help optimizing the contribution of this manuscript to the literature.

We would like to thank the reviewer for the assessment and comments regarding our manuscript.

1. The RML model is stated to be bioinspired and individual modules are linked to certain brain regions. For the dACC (MPFC) this is obvious and needed to create the link to the fMRI results. Also the involvement of the LC and VTA appear reasonable. However, it might be necessary to state that a direct test of the assumptions of the roles of LC and VTA are still pending (for example, through systematic pharmacological challenge studies).

Although we based the design of the RML on neuroscientific literature (e.g. regarding the MPFC-brainstem connections and the role of LC in effort, see Silvetti et al., 2018), we agree that, to the best of our knowledge, there are no studies yet that provide direct evidence for the roles of LC and VTA as hypothesized by the RML. As we agree that this is an important step in future research, we have added a paragraph in the Discussion section ("Limitations and future directions") reporting this issue.

Also, as a minor remark, I was surprised that only LC has an influence of the decision boundary in the DDM, given that dopamine has a clear effect on response vigor.

In the RML we modeled only the mesolimbic DA system, ignoring the mesocortical component (see Silvetti et al. 2018, 2023 for an extensive discussion about this) that would probably be directly involved in the modulation of the DDM. This is a limitation due to minimizing the model complexity. However, in the RML, DA impacts vigor as well, because DA influences policy optimization about top-down modulation of LC activity (boost). For example, in Silvetti et al. 2018 and Caligiore et al. 2020, we showed how a simulated disruption of the mesolimbic DA triggers a cascade effect in the MPFC that results in LC dysregulation, and thence, in a deficit of effort allocation. For this reason, we advance the hypothesis that the role of mesolimbic DA in vigor modulation may be also indirect.

2. I thin that some further elaboration on the putative common representation of cognitive control and monitoring variables in dACC would be helpful. Are these two sets of variables and computations assumed to be carried out by the same neuronal ensembles? It might be worth discussing this in the light of findings that representations in the dACC and adjacent regions are multiplexed (e.g., Fu et al., Science, 2022; Kennerley et al., 2011, Nat Neurosci).

The RML is a system-level model, therefore we are agnostic about the size and the distribution of MPFC neural populations coding for different variables (surprise, expected value, cognitive control,

etc.). We agree that it is an important aspect, therefore we included a brief paragraph about this topic in the Discussion, before the “Limitations and future directions” section.

Also, it might be important to point out the timing of the two functions. While in fMRI studies the different activity increases related to monitoring and control, respectively, seem to overlap in a common activity pattern, EEG, MEG, and intracranial recordings can reveal a temporal dissociation. Can the RML make clear and testable predictions for time-resolved studies?

This is an important point. While the RML can make some testable predictions for time-resolved studies, it can only do so in a limited way, because the current version of the model is implemented within the discrete MPD paradigm (therefore we have a low time resolution). In the revised version of the manuscript, we discuss this topic in the “Limitations and future directions” section.

3. very minor: Figure caption in Fig 3 refers to panel g which does not exist.

The manuscript structure has been extensively revised, including all the figure captions.

Response to reviewer 3

Reviewer #3: In this manuscript, Vriens et al. compare simulations from a Meta-Reinforcement Learning model to fMRI data from two tasks that involve cognitive control. These tasks are selected because results could not be explained by a single existing model. In contrast, the Reinforcement Meta-Learner (RML) model successfully captures neuroimaging results for both tasks. The authors focus on activity in dorsal anterior cingulate cortex (dACC), as the role of this region has been particularly debated in previous work, and activity patterns are well explained by the RML model.

The work addresses important questions about the neural and computational basis of cognitive control and the role of dACC specifically. Providing evidence for a model that unifies previously conflicting findings is an important advance in the field. The modelling seems robust, with additional control or replication analyses provided. I have some suggestions that I hope are helpful to further improve the manuscript.

We would like to thank the reviewer for the assessment and comments regarding our manuscript.

1. The authors present convincing visual evidence that the RML simulations closely capture the dACC activity patterns from the original fMRI results in both studies. They additionally provide statistical evidence that the same pattern (quartic, linear) best fits both the fMRI measured and RML simulated activity. I wondered whether there are more direct ways to show that the RML simulations are the best fitting model of the fMRI activity? It would also be helpful if possible to include fit statistics to show the badness or lack of fit in the relevant places, for example surprise and expected value for the verbal VM task, potentially using Bayesian statistics to show a lack of association.

We acknowledge that providing quantitative measures and relative comparisons of linear and nonlinear trends in the RML predictions is desirable. For this reason, we included, for each of the RML predictions, also quantitative metrics relative to the fitting trends and (when needed) an AIC-based comparison between different predictions.

2. While the findings shown in the figures are convincing, the figures and legends could be improved:
a. The text on Figure 3a, 3b etc is very small

The manuscript structure has been extensively revised -based on the Reviewer's suggestion- including all the figure captions.

b. It might be helpful to combine Figure 3a with 3b and combine Figure 5a with 5b, even if separate y axes are required, to show how well the simulations capture activity patterns more directly. This point seems more relevant to the main argument of the paper than the fit of the quartic functions for Figure 3. If the authors decide to keep the quartic models in Figure 3, the linear trends could be added to Figure 5 for consistency.

Please, refer to the previous answer.

c. It is unclear what the meaning or interpretation of Figure 3e is.
d. The figure legends would benefit from additional detail throughout. Figure 3 and 5 specifically

would also be easier to interpret with links to the main findings for example that they support the RML model, surprise is not sufficient to capture pattern etc.

We revised all the figure captions.

3. I think changes to the structure of sections in the manuscript could help highlight the key contributions of the work. The results section contains a lot of methodological information and the methods section at the end refers to the supplementary information. It seems it may be possible instead to have a methods section between the introduction and results with the sections describing the model and tasks. Then the results section can get into the key findings much sooner.

As anticipated above, we deeply revised the manuscript structure, following the Reviewer's suggestion.

4. In addition to the structure, the writing could also better highlight the key findings in a way that is easier for readers less familiar with the exact predictions of different models. For example, starting results sections with the specific aim of that analysis, relative to the overall aim of the paper, and using 'signposting' phrases such as "As expected if the RML model captures… we found...".

Although we believe that reordering the Methods and Results improved this already, we have additionally made some minor edits to the writing, as requested.

5. The number of participants simulated for the speeded task or whether results are averaging across participants is not clear.

While this information was available in the Supplementary Material, we agree that this information was not available in the main text. We have added this information in the Methods section as well.

6. There seems to be inconsistencies between the main text and supplement on whether there are 3 components in 2 modules or 3 modules. If some quantifications are specific to MPFC activity or another subdivision this could be clearer.

We would like to thank the reviewer for the comment and regret that this has caused any confusion. We have made some changes to the main text to make it more in line with the more detailed description in the supplementary materials and the explanations of the RML in previous literature (Silvetti 2018).

7. The authors clearly acknowledge that equating the boost module in the model with cognitive control is a simplification, could the discussion include a short section on the implications of this?

We think the Reviewer refers to the RML description section. There we meant to highlight that the boost signal modulates not only effort (cognitive or physical) but it also modulates motivation, by influencing the mesolimbic DA output. In that sense, equating the boost to cognitive control is a simplification. We revised the sentence to make it clearer.

8. The discussion highlights the RML-C as an improvement to the RML. Could this section also state whether the differences between these versions of the model would impact the findings of the current paper? This could include suggesting how future work might examine the association between intrinsic motivation and cognitive control, based on the importance of task design and selection as highlighted in this work.

We agree, please refer to the answer to Reviewer #1, Point 4.

Minor:

1. The following sentence is very long, splitting it would help clarity: "The dorsal anterior cingulate cortex (dACC) and the surrounding cortical areas within the medial prefrontal cortex (MPFC) are known to be involved in cognitive control processes (e.g. 6), as well as in Reinforcement Learning (RL) and decision-making (e.g. 7), and the underlying computational mechanisms are still unclear, above all considering that their activation has been routinely found in many different experimental paradigms investigating several aspects of higher-order cognition8–19"

2. As this section is comparing the two existing frameworks, the "however" in the middle is a bit confusing as it comes before further evidence supporting EVC. "A growing body of literature reports activity in the dACC linked to cognitive control (or more generally during anticipation of cognitive effort), in keeping with the perspective of the EVC framework21 (see also 27,28). However, given that in some of these cognitive control studies dACC activity is observed in the absence of surprise or prediction errors3,24,29 or while controlling for all possible sources of surprise30,31, these findings are not easily accommodated by the performance monitoring framework."

We would like to thank the reviewer for these two minor comments and have made some changes to the text to improve the readability of the text.
